# On-Chip Mid-Infrared Wavefront Sensing Based on Vectorial Photocurrent Manipulation

**DOI:** 10.3390/s26134022

**Published:** 2026-06-24

**Authors:** Tao Ye, Xiaofei He, Jun Ning, Xueling Guo, Xianda Zhang, Ziao Li, Wei Lu, Xiaoshuang Chen, Jing Zhou

**Affiliations:** 1 Shanghai Institute of Technical Physics, Chinese Academy of Sciences, Shanghai 200083, China; yetao01@mail.ustc.edu.cn (T.Y.); hexiaofei24@mails.ucas.ac.cn (X.H.); ningjun24@mails.ucas.ac.cn (J.N.); guoxl@mail.ustc.edu.cn (X.G.); zhangxianda25@mails.ucas.ac.cn (X.Z.); liziao25@mails.ucas.ac.cn (Z.L.); luwei@mail.sitp.ac.cn (W.L.); 2 University of Chinese Academy of Sciences, Beijing 100049, China

**Keywords:** wavefront sensing, mid-infrared, graphene, photo-thermoelectric effect, on-chip integration, phase imaging

## Abstract

Wavefront sensing (WFS) is fundamental to adaptive optics, astronomical observation, biological microscopy, and free-space optical communications. However, conventional approaches—including Shack–Hartmann sensors, shearing interferometers, and transport of intensity equation-based methods—are inherently limited by trade-offs among spatial sampling density, angular dynamic range, and device compactness and have rarely been extended to the mid-infrared range. Here, we propose an on-chip mid-infrared wavefront sensing scheme operating based on vectorial photocurrent manipulation and analyze the properties of the proposed device through finite-element simulations. The proposed device comprises a hexagonal array of antenna-integrated graphene pixels, each equipped with three contacts and a microlens. Based on the antenna-induced vectorial photocurrent manipulation, angle-dependent absorption is translated into photocurrent signals, potentially enabling unambiguous recovery of both the elevation and azimuth angles of the incident light over an effective angular dynamic range of ±28°. The hexagonal layout provides a high spatial sampling density of 11,547 mm^−2^. Southwell algorithm-based wavefront reconstruction and numerical simulations yield faithful recovery of parabolic, conical, and quadrangular pyramidal wavefronts. In addition, simulation results indicate that this approach can enable high-fidelity reconstruction of both the phase and intensity distributions of an object based on angular-spectrum diffraction theory. Overall, this work theoretically demonstrates a new route toward high-density wavefront measurement and complex light field imaging in the mid-infrared range without a conventional imaging lens.

## 1. Introduction

In addition to intensity [1], polarization [2,3,4,5], and wavelength [6,7], the wavefront profile represents a critical dimension of the light field, corresponding mathematically to its spatial phase distribution. Phase information encodes key physical quantities such as the depth profile, the morphology features, and the refractive-index distribution of a target and therefore represents an essential information dimension for improving optical imaging performance and environmental sensing capability. Wavefront sensing (WFS) aims to quantitatively capture the phase (or phase gradient) information within a specific plane and plays a fundamental role in adaptive optics [8], astronomical observation [9], biological microscopy imaging [10], and free-space optical communications [11].

As modern optical systems continue to advance toward higher compactness, wider field of view, enhanced spatial resolution, and stronger robustness against disturbance, wavefront sensing faces unprecedented challenges in achieving high spatial sampling density (spatial resolution), large angular dynamic range, and high-level integration. Unlike radio-frequency signals, optical oscillation frequencies are on the order of hundreds of THz, far beyond the direct sampling capability of existing electronics. Therefore, wavefront information is typically acquired indirectly through interference-induced intensity modulation [12], and phase is recovered using inversion algorithms [13].

**Figure 1:**
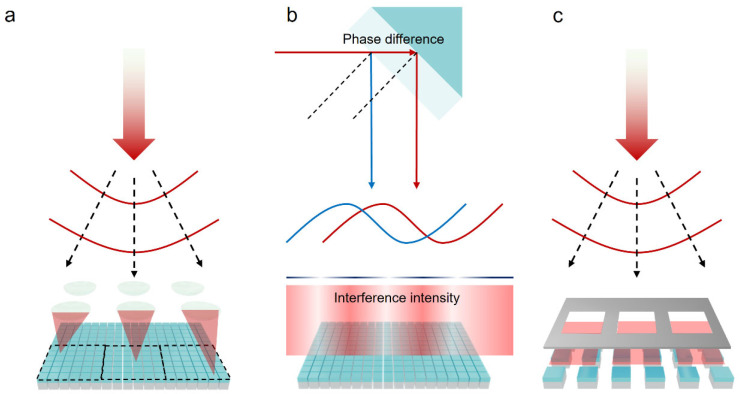
Schematic of wavefront sensing schemes. (**a**) Shack–Hartmann method: wavefront tilt at the aperture is inferred from focal spot displacement; (**b**) shearing interferometry: wavefront is inferred from self-interference fringes of two sheared beams; (**c**) on-chip near-field interference method: local tilt and wavefront are inferred from multi-terminal response ratios of subwavelength microstructures.

Figure 1 schematically compares three representative wavefront-sensing paradigms. Reviewing the development of wavefront sensing technologies, a representative scheme in the geometrical-optics paradigm is the Shack–Hartmann wavefront sensor (SHWFS) [14,15,16,17,18]. As shown in Figure 1a, SHWFS features a relatively simple structure, high measurement accuracy, and convenient real-time implementation, and has thus far remained the prevailing solution for wavefront sensing. However, such systems inevitably suffer from low spatial sampling density (typically ~100 μm) and small angular dynamic range (typically ~1°), making it difficult to meet the demand for high-density measurement of high-order aberrations under wide-field conditions.

Figure 1b shows the basic framework of the interference scheme; interferometric schemes (e.g., shear interferometers) can achieve subwavelength-scale high-spatial-resolution wavefront measurements [12,19,20]. However, they typically rely on long optical paths in free-space configurations, making them highly sensitive to mechanical stability and alignment errors. In addition, their large system footprint and structural complexity limit on-chip implementation.

Wavefront detection techniques based on the transport of intensity equation (TIE) exploit the constraint relationship between phase and intensity evolution along the propagation direction [21]. By collecting intensity distributions at different defocus planes and performing phase retrieval, wavefront information can be obtained. However, such methods often require mechanical scanning along the propagation direction or switching among multiple planes, which increases measurement latency and is intrinsically disadvantageous for compactness and on-chip integration.

Against this backdrop, on-chip wavefront sensing based on near-field interference is regarded as a promising route to meet the demand for highly integrated wavefront measurement [22,23,24,25,26]. As shown in Figure 1c, when optoelectronic structures are reduced to subwavelength scales, light undergoes significant near-field interference before being absorbed and converted into electrical signals [27,28,29,30]. By designing micro/nanostructures that map the local propagation direction of the optical field (i.e., local wave-vector/momentum information) to differences in multi-pixel photocurrents, the local phase gradient can be inferred from wavelength-scale pixel-level photocurrent, enabling reconstruction of the full wavefront. Previous studies have demonstrated the capability of such schemes to detect incident angles/phase gradients in the visible-to-near-infrared range [22,24]. Moreover, monolithic integration with CMOS detector arrays has been implemented to enhance both spatial resolution and angular dynamic range, achieving a spatial sampling density of 9246 mm^−2^ [25].

Nevertheless, existing on-chip integrated wavefront sensors are confined to the visible-to-near-infrared range. Meanwhile, the mid-infrared spectral range is of particular importance for applications including combustion diagnostics, astronomical observation, and semiconductor material inspection. Extending the existing on-chip wavefront-sensing strategies to the mid-infrared range remains challenging. A direct geometric scaling of sensor dimensions from 0.65 μm to 4.75 μm would reduce the spatial sampling density from 9246 mm^−2^ to approximately 173 mm^−2^, leading to a 53.4-fold times of reduction in spatial resolution. Moreover, even the most structurally compact schemes still require four or more independent readout pixels to determine the local tilt, which is unfavorable for further improvements in pixel density and array size.

To address this issue, we propose a mid-infrared wavefront sensor based on vectorial photocurrent manipulation. Each pixel of the wavefront sensor is formed by a piece of graphene with three contacts, each of which is integrated with an antenna. The antenna-induced spatial modulation of both the Seebeck coefficient and the photothermal temperature profile enables the photo-thermoelectric (PTE) current to be selectively routed to different terminals according to the transverse wave-vector components *k_x_* and *k_y_* of the incident light [31,32,33,34]. While maintaining a compact structure, this scheme is projected to achieve a spatial sampling density of 11,547 mm^−2^ and an angular dynamic range of ±28°, projected to outperform previously reported on-chip wavefront sensing schemes in key metrics and potentially providing a new pathway for high-density, large-dynamic-range wavefront measurement in the mid-infrared range.

## 2. Optical Simulation of the Proposed Device

**Figure 2:**
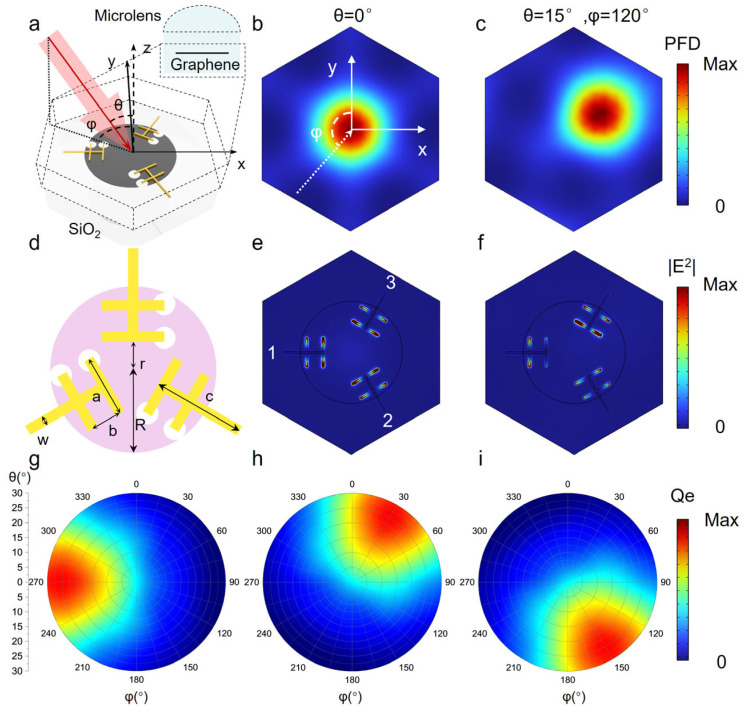
Device structure and optical simulations. (**a**) Three-dimensional pixel structure and definition of incident angles; (**b**,**c**) power flow density distributions in the graphene plane without gold antennae; (**d**) contacts of the graphene nanodisk and the three antennae integrated; (**e**,**f**) local field intensity distributions after introducing gold antennae; (**g**–**i**) electromagnetic losses *Q*_e1–3_ of the three electrodes versus incident tilt angles.

The device scheme is schematically depicted in Figure 2a (detailed geometric parameters are provided in SI Note S1). The graphene–gold optical antenna/electrode integrated PTE pixel is packaged within a microlens on a SiO_2_ substrate. The three gold electrodes are independently routed outward. In addition to collecting photocurrent, they also serve as optical antennae that modulate the local absorption. The array adopts a regular hexagonal periodic arrangement, with unit side length W = 5 μm. For a regular hexagonal unit cell, the cell area is (3√3/2)W^2^ = 64.95 μm^2^. Assuming a fill factor of 75% (accounting for inter-pixel gaps and routing area), the effective spatial sampling density is 0.75/64.95 μm^2^ ≈ 11,547 mm^−2^. An anticipated fabrication process is provided in SI Note S2. For convenience, starting from the left electrode and numbering counterclockwise, the electrodes are labeled 1–3.

The local wavefront tilt of the incident light is described by the elevation angle *θ* and the azimuth angle *φ* (schematically defined in Figure 2a). With these two angles, the transverse wave-vector components *k_x_* and *k_y_*—i.e., the transverse phase gradients—of the incident light write (see SI Note S10 for a schematic diagram):

(1) $$k_{x}=k\cdot sin(\theta )cos(\varphi )$$

(2) $$k_{y}=k\cdot sin(\theta )sin(\varphi )$$

where *k* = 2π/λ. The normalized wavefront gradient components are defined as *K_x_ = k_x_/k* = *sin*(*θ*)*cos*(*φ*) and *K_y_ = k_y_/k* = *sin*(*θ*)*sin*(*φ*). To clarify the wavefront sensing mechanism, we first analyze the role of the microlens without introducing the gold antennae. The simulation of microlenses was conducted using frequency domain FEM. The complete 3D power flow density (PFD) cross-sectional diagram is presented in SI Note S7. Figure 2b,c show the PFD distribution in the graphene plane under illumination by unpolarized light at *θ* = 0° and *θ* = 15°, *φ* = 120°. Similar to a conventional lens, the microlens maps the local incident tilt angle in momentum space to a displacement of the focused light spot in real space. When *θ* = 0°, the power flow density exhibits a highly symmetric focused distribution near the geometric center of the pixel (Figure 2b). When *θ* ≠ 0°, oblique incidence causes a shift in the focused light spot, with the shift direction determined by the azimuth angle *φ* (Figure 2c). Therefore, if the focused light spot position can be effectively encoded in the photocurrent, the local direction of the incident light can be determined. It should be noted that the periodic arrangement of the array means that, with increasing incident angle, the optical coupling from neighboring unit cells gradually becomes non-negligible. This effect may lead to aliasing in the determination of the focal spot position, thereby constraining the achievable dynamic range. In the following, we quantitatively evaluate the effective angular dynamic range of the proposed device. This range is defined by the condition that the wavefront tilt can be uniquely determined from the photoresponse.

Figure 2d depicts the graphene nanodisk and the three antenna-integrated contacts. Each antenna consists of two short strips oriented along the azimuthal direction and one long strip extending along the radial direction. The full-wave simulation results are shown in Figure 2e,f. The antennae enhance the local field at the tips. At *θ* = 0°, the field intensity at the inner strip of the annular antenna is higher than that at the outer strip, reflecting light focusing due to the microlens; under oblique incidence, the shift in the focused light spot induces systematic changes in the local field intensity distribution. The antenna closer to the light spot center exhibits a higher local field intensity. Since the photocurrent is strongly correlated with the rise in local absorption/temperature [35,36,37], it is expected that the local wavefront tilt angle information can be encoded in the magnitudes and relative relationships of the photocurrents collected by the three contacts.

To more clearly reveal the influence of tilt angle on antenna absorption, we calculate the electromagnetic losses *Q*_e1_, *Q*_e2_, *Q*_e3_ of the three gold antennae as functions of *θ*, *φ* (Figure 2h,i). Owing to the 120° rotational symmetry of the three antennae, their angular loss functions *Q*_e_ (*θ*, *φ*) exhibit a translational correspondence in the azimuthal domain, with mutual offsets of 120°. The maximum antenna absorption occurs near *θ* ≈ 25°, and the corresponding *φ* at the peak has a definite geometric relationship with the electrode azimuth, providing a monotonic/invertible region exploitable for subsequent analytical inversion.

## 3. Optoelectronic Simulation and the PTE Mechanism

**Figure 3:**
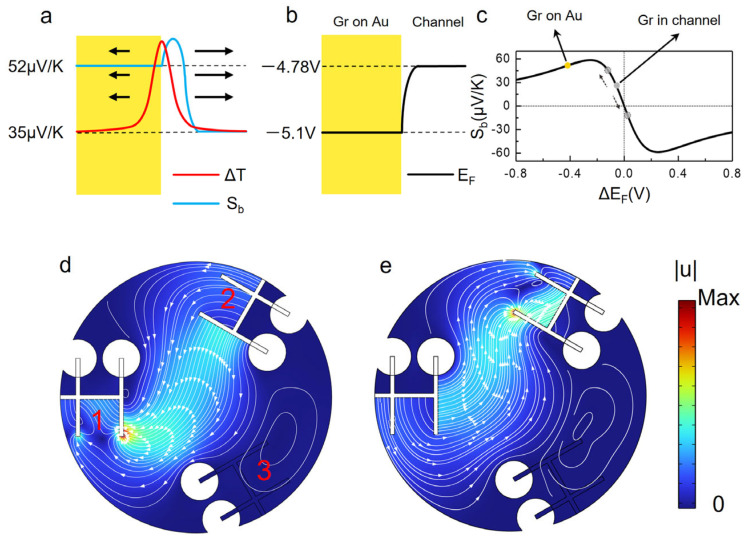
PTE mechanism and optoelectronic simulations. (**a**) Schematic of PTE photoresponse: local carrier temperature rise together with nonuniform S_b_(**r**) generates a net driving force; (**b**) Fermi-level bending induced by graphene–electrode contact; (**c**) *S_b_*(*E_F_*) relationship; (**d**,**e**) carrier transport simulations: electrode 1 absorption *Q*_e1_ contribution and electrode 2 absorption *Q*_e2_ contribution.

The photoresponse of graphene–gold integrated optoelectronic devices in the mid-infrared band is commonly attributed to the photo-thermoelectric (PTE) effect [31,38]. As illustrated in Figure 3a, the PTE effect originates from the near-field optical field localization function of the gold antenna, which locally heats the carriers in graphene and thereby establishes a temperature gradient. When a position-dependent Seebeck coefficient distribution, $S_{b}(r)$, exists in graphene, the temperature gradient drives a net directional carrier transport, resulting in a measurable macroscopic photocurrent.

Given that the characteristic size of graphene in this work (~5 μm) is much larger than the carrier ballistic transport length (~50 nm), carrier transport can be described using a viscosity-dominated “fluid-like” model, whose steady-state equation can be written as

(3) $$F_{PTE}(r)+\mu \;\nabla^{2}u(r)-\beta \;u(r)=0$$

where **F**_PTE_ is the PTE driving force term, *μ* is the effective dynamic viscosity of carrier (dominated by electron–electron scattering), *β* is the drag coefficient (related to electron–phonon scattering), and **u** is the local carrier drift-velocity. Parameter extraction and the numerical solution procedure are provided in SI Note S3. The PTE driving force satisfies

(4) $$F_{PTE}\propto -S_{b}(r)\;\nabla T(r)$$

where T(r) is linearly proportional to the local optical field intensity ${\left|E\right|}^{2}$.

The near-field optical localization effect of the gold antennae gives rise to sharp carrier temperature peaks at the antenna edges. If *S_b_*(**r**) is spatially uniform, ∇*T* on both sides of the temperature peak has the same magnitude but opposite direction, leading to the cancellation of the driving forces zero net photocurrent. Therefore, *S_b_*(**r**) must be nonuniform in space to generate a net photocurrent. The work-function difference between gold and graphene introduces Fermi-level pinning at the gold–graphene contact region [39]. This pinning causes spatial bending of the graphene Fermi-level $E_{F}(r)$ near the electrodes. As a result, $S_{b}(r)$, which strongly depends on $E_{F}(r)$, becomes spatially nonuniform. The spatially nonuniform *S_b_*(**r**) breaks the symmetry protection, so that a directional net photocurrent can be generated in the device (Figure 3a,b).

The relationship between *S_b_* and *E_F_* can be approximately described by the Mott formula [40]:

(5) $$S_{b}(E_{F})=-\frac{\pi^{2}\;k_{B}^{2}\;T}{3e}\cdot \frac{1}{\sigma (E_{F})}\cdot \frac{d\sigma (E_{F})}{dE_{F}}$$

(6) $$\sigma (E_{F})=\sigma_{min}\;\left(1|\frac{E_{F}^{2}}{\Delta^{2}}\right)$$

where *k_B_* is the Boltzmann constant, *e* is the elementary charge, *σ*_min_ is the minimum conductivity, and Δ characterizes the width of the charge-neutrality region (in this work, Δ = 0.25 eV); the *S_b_* (*E_F_*) curve is shown in Figure 3c.

We first obtained the simulated $|E{|}^{2}$ distribution from full-wave optical simulations. Then, we performed carrier fluid-transport simulations based on this optical field distribution, together with the graphene geometry and the electrode boundary conditions. Figure 3d shows a typical result under a normally incident optical field; the locally enhanced optical field at the antenna tips drives directional carrier transport. Under normal incidence, mirror symmetry suppresses the net photocurrent for all electrode-pair readout schemes, leading to a degenerate response. To lift this degeneracy, a hole is introduced in the graphene near one end of the vertically aligned antenna stem, thereby explicitly breaking the symmetry and allowing a nonzero photocurrent to be detected at normal incidence (for example, forming net transport from electrode 3 toward electrode 1, yielding *I*_31_).

The measured photocurrents under connections of electrode pairs 1–2, 2–3, and 3–1 are defined as *I*_1_, *I*_2_, and *I*_3_, respectively. To determine the electromagnetic loss at each electrode and then solve for the local incident direction, it is essential to establish a relationship between the measured photocurrent and electromagnetic loss *Q_e_*_1–3_. Through a control simulation that “retains only the local absorption contribution of a single electrode” (Figure 3e), current decomposition coefficients can be obtained from the carrier hydrodynamic transport simulations (velocity field shown in Figure 3d,e). By integrating the carrier flux collected at each terminal, the following linear relations are established:

(7a) $$I_{1}=Q_{e1}-1.188\;Q_{e2}$$

(7b) $$I_{2}=Q_{e2}-1.188\;Q_{e3}$$

(7c) $$I_{3}=Q_{e3}-1.188\;Q_{e1}$$

The numerical coefficient 1.188 in Equation (7) is derived from the carrier hydrodynamic simulation (see Figure 3d,e). Its specific calculation procedure is as follows: assuming separately that electrodes 1 and 2 each have the same optical absorption *Q*_e_, calculate the carrier flux from electrode 1 to terminal 2. When electrode 1 has light absorption and electrode 2 has light absorption, the current *I_a_* and the photocurrent *I_b_* correspond to the contributions of the light absorption of electrode 1 and electrode 2, respectively, to the total photocurrent. The value of 1.188 is derived from the ratio of *I_b_* and *I_a_* obtained in the simulation. When *I*_1_, *I*_2_, and *I*_3_ are obtained, solving the above system of three linear equations yields *Q*_e1_, *Q*_e2_, and *Q*_e3_, laying the foundation for subsequent inversion of the local incident tilt angles from the absorption distribution.

## 4. Wavefront Reconstruction Simulation

As mentioned above, the independent variables to be measured before a local light field can be taken are three: power density S, elevation angle θ, and azimuth angle φ. When Q_e1–3_ are known, the corresponding (S, θ, φ) can be analytically obtained within the angular dynamic range. Since Q_e1–3_ are linearly related to S, to focus on the wavefront tilt (θ, φ) and eliminate the influence of S, we introduce normalized auxiliary variables (the absolute photocurrent magnitudes are proportional to the incident power density S, while the ratios M_1,2_ depend only on θ and φ):

(8) $$M_{1}=log\;\left(\frac{Q_{e3}}{Q_{e1}}\right),M_{2}=log\left(\frac{Q_{e2}}{Q_{e3}}\right)$$

**Figure 4:**
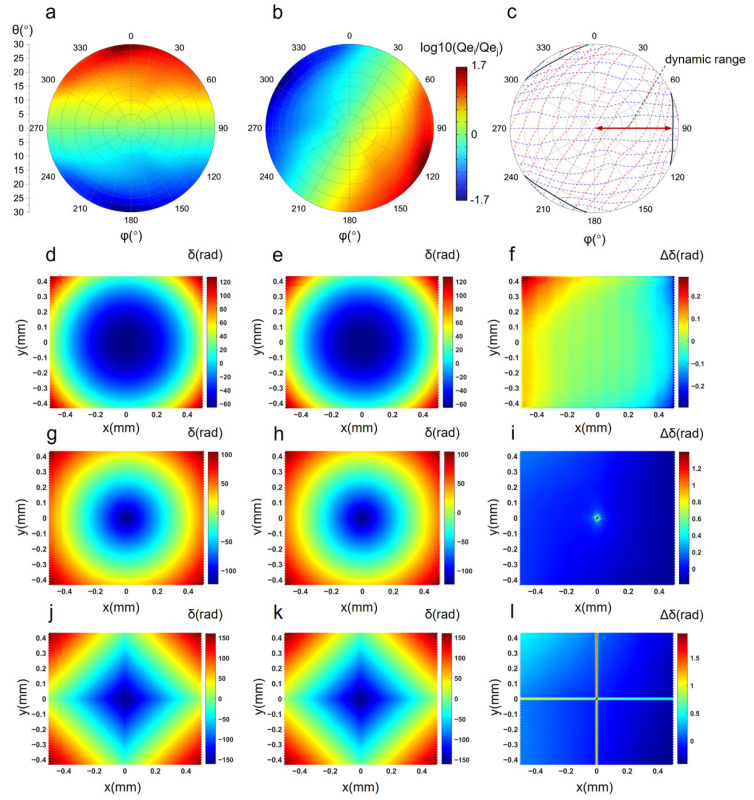
Principles and simulations of wavefront reconstruction. (**a**,**b**) Mapping relationships between M_1_, M_2_ and θ, φ; (**c**) effective dynamic range obtained from contour superposition; (**d**–**l**) reconstruction and error analysis for parabolic, conical, and tetrahedral wavefronts.

Figure 4a,b show the mapping relationships of *M*_1_, *M*_2_ versus *θ*, *φ*: within a certain angular range, the contour lines of *M*_1_ and *M*_2_ exhibit approximately orthogonal monotonic variation characteristics, making it possible to invert *θ*, *φ* from *M*_1_, *M*_2_.

To quantify the effective angular dynamic range of the proposed device, we overlaid the contour maps in Figure 4a,b to obtain Figure 4c. We define the dynamic range of angles as the θ, φ range that can uniquely recover the incident angles θ, φ from *M*_1_, *M*_2_. This requires the red and blue contour lines to intersect transversely rather than tangentially. Tangential intersections indicate local Jacobian degeneracy, which leads to nonunique solutions or ill-conditioned inversion. The black solid line therefore marks the boundary of the angular dynamic range. The fundamental mechanism limiting the angular dynamic range is that, as the incident angle increases, optical coupling between neighboring pixels becomes non-negligible. When the focal spot shifts sufficiently close to a pixel boundary, the electrode absorption ratios *M*_1_ and *M*_2_ are no longer monotonically related to the incident direction, leading to Jacobian degeneracy where *M*_1_ and *M*_2_ contours become tangent rather than transversely intersecting. This boundary defines the effective angular dynamic range of ±28°. A more intuitive way to observe the device’s angular dynamic range is to examine the relationship between the time-averaged power flow density on the graphene plane and the incident angle in the absence of microstructures. As the incident angle increases, optical crosstalk from neighboring pixels causes the time-averaged power flow density to exhibit a rotational symmetry similar to that at normal incidence, thereby making it impossible to uniquely determine the incident angle from the photocurrents; see SI Note S9 for details.

To verify the wavefront sensing capability of the proposed device, we simulated the reconstruction of wavefronts with large phase gradients. Taking a parabolic wavefront as an example (Figure 4d–f), the wavefront aperture is 1 mm × 0.867 mm, and the number of pixels is 100 × 100, corresponding to a spatial sampling density of 11,547 mm^−2^. The maximum phase difference across the entire aperture reaches 190 rad, which corresponds to an optical path difference of about 0.144 mm for *λ* = 4.75 μm; the maximum phase gradient reaches 0.4118 *k*, corresponding to an equivalent incident tilt angle of *θ* ≈ 24.32°.

The wavefront reconstruction procedure is summarized as follows. For each pixel, we first measure the three photocurrents, $I_{12}$, $I_{23}$, and $I_{31}$. We then solve the corresponding linear system (7) to obtain $Q_{e1}$, $Q_{e2}$, and $Q_{e3}$. From these quantities, we calculate $M_{1}$ and $M_{2}$ and determine the incident angles θ and ϕ using the calibrated mappings in Figure 4a,b. The local phase gradients are then obtained from θ and ϕ. Finally, the full wavefront is reconstructed using the Southwell wavefront analysis algorithm [41] (see SI Note S4 for details).

The reconstructed parabolic wavefront at a photoresponse measurement signal-to-noise ratio of 35 dB is shown in Figure 4e—signal-to-noise, the rationale for which is discussed in SI Note S5. It agrees well with the ground truth and with a root-mean-square error (RMSE) calculated point by point of 0.0686 rad, demonstrating the feasibility of the proposed method for wavefront reconstruction. The error distribution shows that errors at the edge of the aperture are significantly higher than those at the center. The increase in error at the edge is attributed to two possible reasons. First, the phase gradients near the edge are closer to the dynamic range boundary, increasing inversion error. Second, the Southwell algorithm can exploit averaging over more neighboring gradient data at the center of the aperture to suppress random noise, whereas the aperture edge has insufficient neighborhood information, leading to increased error.

In addition, the proposed method successfully reconstructed both conical and tetrahedral wavefronts (Figure 4g–l), further demonstrating its capability for wavefront reconstruction. For the conical wavefront, the phase remains continuous over the full aperture, whereas the phase gradient is undefined at the vertex. For the tetrahedral wavefront, the phase gradient is similarly undefined along the ridge edges, leading to increased reconstruction errors in these regions. Among all the tested cases, the tetrahedral wavefront exhibits the largest overall RMSE of 0.2463 rad, owing to its greater number of gradient singularities.

To analyze the robustness of the proposed design under practical fabrication errors, we examined the influence of geometric dimension errors and local contact resistance fluctuations. Among geometric dimension errors, the graphene material and metal antenna structures are fabricated using UV lithography and electron-beam lithography, respectively, with resolutions and overlay accuracies on the order of tens of nanometers to several nanometers, so their geometric errors are negligible. The most significant geometric fabrication error is expected to arise from the imprint alignment error of the microlenses. The overlay alignment accuracy of current commercial nanoimprint lithography processes is less than 500 nm (NPS300 model); accordingly, we analyzed the impact of imprint lens misalignment of 500 nm in each of two orthogonal directions on the proposed device. The results indicate that under 500 nm imprint alignment deviation, the fundamental properties of the proposed device are preserved, with the angular dynamic range slightly decreasing from 28° to 23°. The proposed device remains functional; detailed results are presented in SI Note S8.

Local contact resistance fluctuations originate from the nonuniformity at the graphene–metal electrode contacts. The device resistance consists of two components: the channel graphene resistance and the contact resistance. Without annealing, the graphene–metal contact resistance can be set to 43 kΩ·μm, with a fluctuation range of ±5 kΩ·μm, corresponding to a relative fluctuation of approximately 8% [42]. Since the micrometer-thick PC microlens layer serves as an encapsulation layer, the contact resistance can remain stable over extended periods [43,44,45,46,47]. Contact resistance variations can be corrected through pre-calibration; thus, electrical contact nonuniformity likewise will not significantly degrade the performance of the proposed device.

## 5. Phase Imaging Without Conventional Imaging Lens Simulation Based on Wavefront Measurement

An important application of wavefront sensing is phase imaging without conventional lenses. In conventional lens-based imaging systems, image quality is fundamentally limited by the modulation transfer function of the optics. Defocus aberrations further degrade image quality [48]. When imaging objects with pronounced three-dimensional surface variations or under severe defocus, increasing the numerical aperture to enhance lateral resolution often comes at the expense of depth of field. As a result, high-spatial-frequency components are attenuated by defocus, leading to the loss of fine image details [49,50,51].

**Figure 5:**
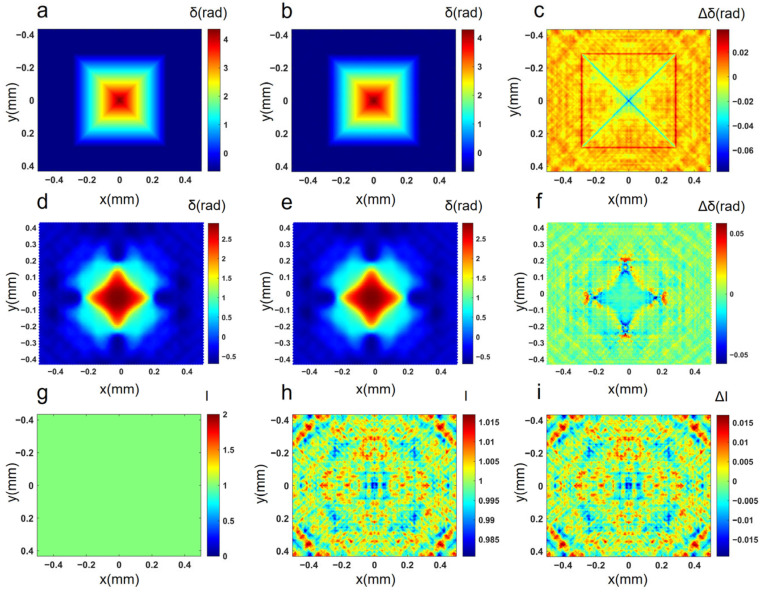
Phase imaging simulation. (**a**–**c**) Ground truth, reconstructed, and error maps of the tetrahedral wavefront in the object plane, RMSE = 0.0078 rad; (**d**–**f**) ground truth, reconstructed, and error maps of the wavefront in the image plane; (**g**–**i**) ground truth, reconstructed, and error maps of the light intensity in the object plane.

Wavefront sensing provides phase information at the detection plane. Under near-paraxial conditions, using angular-spectrum diffraction theory [52,53,54], the complex optical field at upstream object planes can be reconstructed, thereby enabling imaging without a conventional imaging lens. Figure 5 demonstrates phase imaging based on wavefront measurement. The object-plane light intensity was set to 1, and the object-plane phase was a tetrahedral protrusion (Figure 5a). The image-plane wavefront after 1 cm propagation was calculated using angular-spectrum diffraction theory (Figure 5d) and compared with the measured result (Figure 5e). The discrepancy, shown in Figure 5f, is larger in regions with higher local phase gradients. By back-propagating the measured image-plane wavefront by −1 cm based on angular-spectrum diffraction theory, we recovered the object-plane wavefront and intensity distributions (Figure 5b,c,h,i). The reconstructed phase matches the ground truth well, and the recovered intensity remains close to 1, confirming the feasibility of the proposed imaging strategy without a conventional imaging lens.

## 6. Conclusions

In summary, we propose and theoretically investigate an on-chip integrated wavefront detection scheme for the mid-wave infrared. By exploiting the PTE effect to generate vector photocurrents in graphene within a three-terminal integrated graphene–gold antenna structure, the proposed device is projected to simultaneously achieve a large angular dynamic range and a high spatial sampling density, reaching ±28° and 11,547 mm^−2^, respectively. These attributes make the proposed scheme highly promising for applications such as combustion diagnostics, astronomical imaging, and semiconductor material inspection, particularly for dense wavefront sensing in the presence of high-order aberrations, severe distortions, and large phase gradients.

Furthermore, the PTE-driven vector photocurrent mechanism in graphene can potentially enable reliable quantitative phase imaging in the mid-wave infrared, opening a new route toward three-dimensional structural reconstruction.

## Data Availability

The data presented in this study are available upon request from the corresponding author due to privacy considerations.

## Supplementary Materials

The following supporting information can be downloaded at: https://www.mdpi.com/article/10.3390/s26134022/s1.

## Abbreviations

The following abbreviations are used in this manuscript:

WFS	Wavefront sensing
PTE	Photo-thermoelectric
RMSE	Root-mean-square error
SHWFS	Shack–Hartmann wavefront sensor
PFD	Power flow density
