# Supplementary material for: On-Chip Mid-Infrared Wavefront Sensing Based on Vectorial Photocurrent Manipulation
Source: Sensors (Basel). 2026 Jun 24;26(13):4022. doi: 10.3390/s26134022 (PMC13363727; doi:10.3390/s26134022)
Supplement: Supplementary file 1 [file sensors-26-04022-s001.zip › sensors-4360872-supplementary.pdf]

Supplementary Information

Note S1. Device Geometry

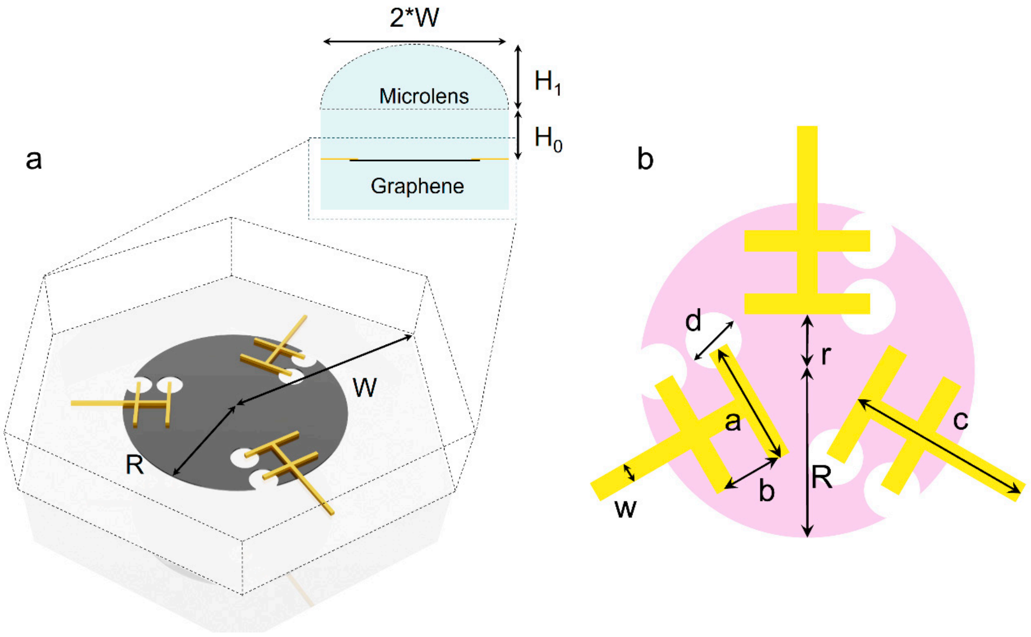

Figure S1. Device geometry. (a) Three-dimensional model of the device. The microlens is a rotationally symmetric parabolic surface. The geometric parameters are W = 5 μm, R = 2.5 μm, H0 = 3.6 μm, and H1 = 4.6 μm; (b) Cross-sectional view of the graphene-based device. The geometric parameters are R = 2.5 μm, w = 80 nm, gold thickness t = 40 nm, a = 1.44 μm, b = 0.8 μm, r = 1.375 μm, and d = 0.7 μm.

Note S2. Proposed Fabrication Process of the Device

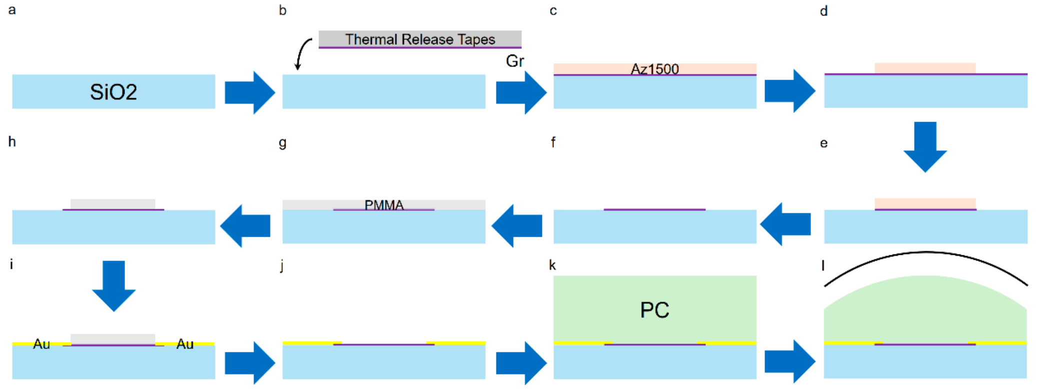

Figure S2. Proposed fabrication process for the device. (a) Prepare the SiO2 substrate.(b) Dry-transfer graphene onto the SiO2 surface using Thermal Release Tapes; (c) Spin-coat AZ1500 UV photoresist; (d) Pattern the resist and develop it; (e) Remove the exposed graphene by plasma etching; (f) Strip the photoresist; (g) Spin-coat PMMA electron-beam resist; (h) Perform electron-beam lithography and development; (i) Deposit the gold electrodes; (j) Lift off the electron-beam resist; (k) Coat the sample with polycarbonate (PC) hot-melt adhesive; (l) Use nanoimprinting to form the microlens profiles.

Note S3. Optoelectronic Simulation Method

The optoelectronic simulations were performed using COMSOL Multiphysics v6.3. The overall workflow is illustrated in Figure S3. The electrostatic and hydrodynamic simulation methodology follows the framework previously established for graphene-based PTE devices[1–3]. The simulation consists of three steps: electrostatic calculation of the graphene contact region, extraction of the spatially varying Seebeck coefficient, and hydrodynamic simulation of photo-thermoelectric carrier transport.

1. Electrostatic simulation

An electrostatic model was first constructed according to the actual device geometry. Graphene was modeled as a two-dimensional conductive sheet with negligible thickness, and the gold electrodes were treated as equipotential boundaries at 0 V. The electrostatic potential V(r) satisfies the Poisson equation:

$$\nabla \cdot (\varepsilon_0\, \varepsilon_r\, \nabla V) \;=\; -\rho_V$$

where ε_0 is the vacuum permittivity, ε_r is the relative permittivity, and ρ_V is the net free-charge density.

The local Fermi-level distribution in graphene was then obtained from the electrostatic solution. In the present model, the Fermi level is related to the local electrostatic potential through:

$$E_F \;=\; -\Phi_{Au} \;+\; \Phi_g \;+\; V$$

where Φ_{Au} = 5.1 eV [4]is the work function of gold, Φ_g = 4.7 eV [5]is the work function of graphene, and V is the electrostatic potential. The contact-induced doping from the SiO2 substrate is represented by an energy shift Δ_g = 0.08 eV [1].

Following the model used in this work, the net charge density associated with the graphene sheet is written as:

$$\rho_V \;=\; m_p\, g_s\, g_v\, e^2\, ((E_F \;-\; \Delta_g)/(2\pi\hbar^2))$$

or equivalently,

$$\rho_V \;=\; m_p\, g_s\, g_v\, e^2\, ((\Phi_{Au} \;+\; \Phi_g \;+\; V \;-\; \Delta_g)/(2\pi\hbar^2))$$

where m_p = 0.03m_e [6]is the effective hole mass used in the simulation, g_s = 2 and g_v =

2 [6]are the spin and valley degeneracies, respectively, e = 1.6 × 10^{-19} C is the elementary charge, and ħ = 1.05 × 10^{-34} J·s is the reduced Planck constant.

The resulting spatial distributions of the Fermi level $E_F(\mathbf{r})$ and the corresponding Seebeck coefficient are shown in Figure S4.

2. Seebeck coefficient extraction

After obtaining $E_F(\mathbf{r})$, the local Seebeck coefficient $S_b(E_F)$ was calculated using the Mott relation:

$$S_b(E_F) = -(\pi^2 {k_B}^2 T)/(3e) \cdot (1/\sigma(E_F)) \cdot d\sigma(E_F)/dE_F$$

$$\sigma(E_F) = \sigma_{min}(1 + {E_F}^2/\Delta^2)$$

Here T = 300 K is the temperature, k_B = 1.38 × 10^{-23} J·K^{-1} is the Boltzmann constant, and Δ = 200 meV [1]is the width of the charge-neutrality region. In this calculation, σmin acts only as a scaling factor and therefore cancels out when only the relative spatial variation of S_b is required.

3. Hydrodynamic carrier-transport simulation

The photoresponse was then simulated using a hydrodynamic carrier-transport model. The steady-state carrier flow satisfies:

$$F_{PTE}(r) + \mu \nabla^2 u(r) - \beta u(r) = 0$$

where u(r) is the carrier drift-velocity field, μ is the viscosity coefficient, and β is the drag coefficient. The local current density is given by:

$$j = -neu$$

where n = 6.68 × 10^{14} m^{-2} [6] is the carrier sheet density and e is the elementary charge. The PTE driving force is taken to satisfy:

$$F_{PTE} \propto -S_b \nabla T$$

where T denotes the local carrier temperature rise induced by optical absorption. In the simulation, the temperature rise is assumed to be proportional to the local optical-field intensity $|\boldsymbol{E}|^2$, obtained from the full-wave electromagnetic simulation.

The drag coefficient is related to the sheet conductivity through:

$$\beta = ne/\sigma$$

where the graphene sheet conductivity is taken as σ = 5 mS [1]. The carrier-velocity distribution obtained from the hydrodynamic simulation is shown in the main text.

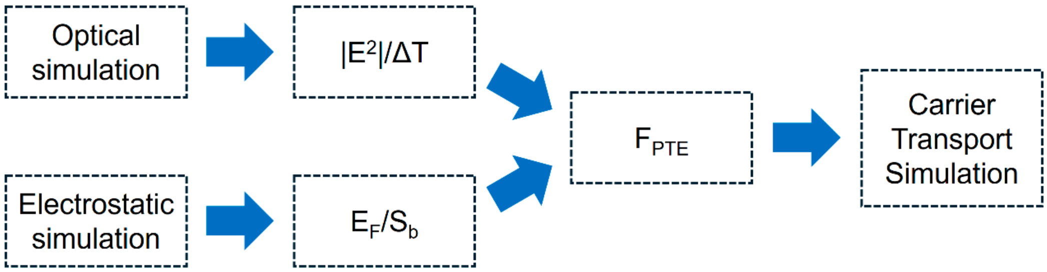

Figure S3. Optoelectronic simulation workflow.

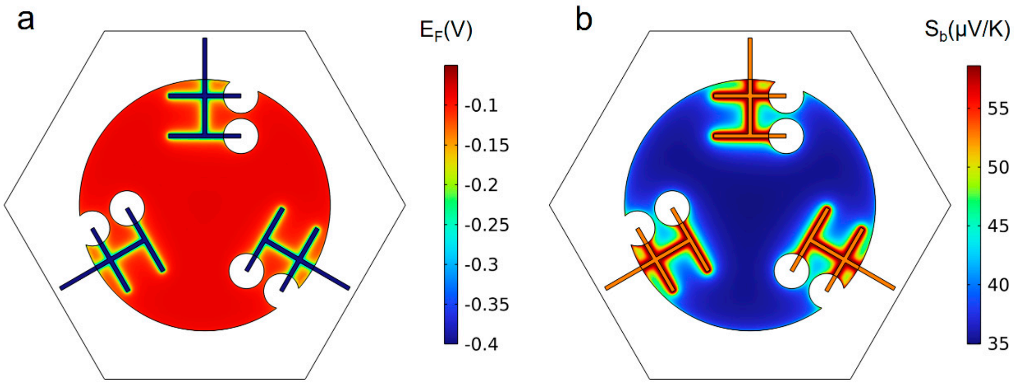

Figure S4. Electrostatic simulation results. (a) Calculated E_F in graphene; (b) Calculated S_b in graphene.

Note S4. Southwell Wavefront Reconstruction on a Hexagonal Lattice

The classical Southwell algorithm [7] was originally formulated for a rectangular lattice, where the phase is defined at grid nodes and the slope is measured at the centroid of each rectangular sub-aperture. In this work, the Southwell method is extended to a hexagonal lattice. Hexagonal microlens arrays can be used in adaptive optics because of their higher fill factor and more efficient sampling than square arrays. To reconstruct the wavefront on a hexagonal lattice, the conventional Southwell method is extended to establish the relationship between the average slope measured within each sub-aperture and the phase values at the surrounding lattice nodes.

1. Geometric model

As shown in Figure S5, the wavefront phase is defined at the vertices of the hexagonal lattice, whereas the local wavefront slope is defined at the centroid of each hexagonal sub-aperture. For a regular hexagonal sub-aperture m with side length W, the six surrounding phase samples are denoted by δ1, δ2, ..., δ6 in counterclockwise order.

2. Discrete slope-phase relationship

Using a least-squares plane-fitting approximation, the x- and y-components of the slope within the m-th sub-aperture can be expressed as weighted linear combinations of the six surrounding phase values. Assuming that one pair of hexagon edges is parallel to the y-axis, the discrete slope operators are written as:

$$\widehat{k_m^x}(\delta) = \frac{1}{3\sqrt{3}W}\left[(\delta_1 - \delta_4) + \frac{1}{2}(\delta_2 - \delta_3) + \frac{1}{2}(\delta_6 - \delta_5)\right]$$

$$\widehat{k_m^y}(\delta) = \frac{1}{3\sqrt{3}W}\left[\frac{\sqrt{3}}{2}(\delta_3 - \delta_5) + \frac{\sqrt{3}}{2}(\delta_2 - \delta_6)\right]$$

3. Global reconstruction formulation

Wavefront reconstruction can then be formulated as an overdetermined linear system:

$$\begin{bmatrix} k_m^x \\ k_m^y \end{bmatrix} = \begin{bmatrix} \widehat{k_m^x}(\delta) \\ \widehat{k_m^y}(\delta) \end{bmatrix}$$

Collecting all slope measurements over the aperture yields the global matrix equation:

$$\boldsymbol{C}\delta = \boldsymbol{K}$$

where δ is the vector of unknown phase values at the lattice nodes, C is the discrete difference matrix, and K is the measured slope vector.

The phase is obtained by minimizing the least-squares error:

$$min\big(Error(\delta)\big) = min(\|\boldsymbol{C}\delta - \boldsymbol{K}\|^{\boldsymbol{2}})$$

$$= min(\delta^T \boldsymbol{C}^T \boldsymbol{C}\delta + \boldsymbol{K}^{\boldsymbol{T}} \boldsymbol{K} - \boldsymbol{2}\delta^T \boldsymbol{C}^T \boldsymbol{K})$$

Setting the derivative with respect to δ equal to zero leads to the normal equation:

$$2\boldsymbol{C}^T \boldsymbol{C}\delta - 2\boldsymbol{C}^T \boldsymbol{K} = \boldsymbol{0}$$

$$\boldsymbol{C}^T \boldsymbol{C}\delta = \boldsymbol{C}^T \boldsymbol{K}$$

Because the phase is defined only up to an additive constant, a zero-mean phase constraint is imposed to obtain a unique solution. In this work, the wavefront is therefore reconstructed by solving the constrained least-squares problem, yielding the phase distribution over the full aperture.

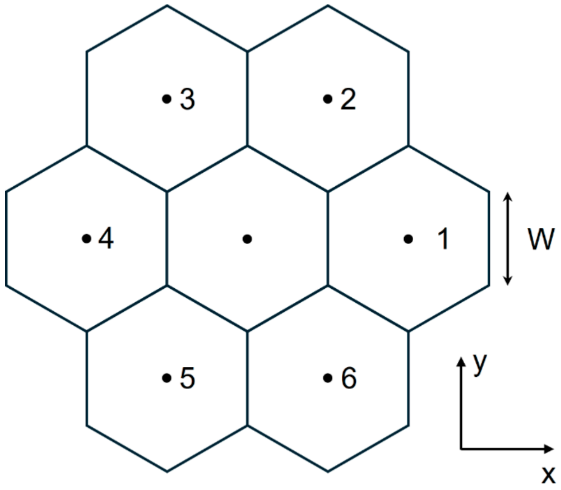

Figure S5. Hexagonal lattice layout and numbering of adjacent nodes.

Note S5. Proposed Device Expected Photovoltaic Performance Analysis

Based on existing reported mid-wave infrared Au-graphene PTE photodetectors, the typical values of the key characteristic parameters are as follows [1–3]: responsivity $R = 53\ \mathrm{mA/W}$ [1], response speed $\tau = 1\ \mathrm{\mu s}$ [2], device area $A = 11\ \mathrm{\mu m^2}$ [1], high-frequency current noise spectral density $i_n = 5.6\ \mathrm{pA\ Hz^{-1/2}}$ [1], and the device noise equivalent power (NEP) is the current noise spectral density divided by the responsivity, namely $\mathrm{NEP} = 1.06 \times 10^{-10}\ \mathrm{W\ Hz^{-1/2}}$, with a detectivity of $D^* = 3.1 \times 10^6$ Jones.

Since the proposed device operates at the same wavelength 4.75 μm as previously reported actual devices and is based on the same physical mechanism, it can be considered to have the same specific detectivity as the typical device. The photosensitive area of the proposed device is $A' = 19.6\ \mathrm{\mu m^2}$ (graphene area), so the NEP increases by a factor of $\sqrt{A'/A} = 1.33$, the final value is $\mathrm{NEP}' = 1.41 \times 10^{-10}\ \mathrm{W\ Hz^{-1/2}}$. At this point, to achieve a signal-to-noise ratio of 35 dB, where the unit bandwidth $f = 1\ \mathrm{Hz}$, we have $\mathrm{SNR} = P^2/\mathrm{NEP}^2 f = 10^{3.5}$, yielding an optical power of $P = 7.9\ \mathrm{nW}$, corresponding to an illumination power density of $7.9\ \mathrm{nW}/64.95\ \mathrm{\mu m^2} = 1.22\ \mathrm{W\ cm^{-2}}$. This power density remains within the applicable range of typical photodetectors and can meet potential application requirements.

As a supplement, we discuss thermal background radiation, Johnson noise, and 1/f noise. Thermal background radiation limits the performance of infrared detectors by setting a background-limited detectivity. For photovoltaic devices, the background-limited specific detectivity at 300 K is $D^*_{BLIP} = 1.8 \times 10^{11}$ Jones[8], which is far higher than the detectivity of the proposed device; therefore, the contribution of thermal background radiation is negligible. Existing literature suggests that the contact resistance of Au-graphene is $75\ \mathrm{k\Omega\ \mu m}$ [9,10]. Through simulation of the inter-terminal current, we obtained the device resistance considering the presence of metal electrode-graphene contact resistance $R = 26.4\ \mathrm{k\Omega}$. At this point, the Johnson noise value at 300 K is $0.79\ \mathrm{pA\ Hz^{-1/2}}$. As for 1/f noise, its general form is: $I_0/\sqrt{f}$ [11], the reported current noise spectral density at 60 Hz is $0.2\ \mathrm{nA\ Hz^{-1/2}}$, so the

relationship between 1/f noise current spectral density and frequency satisfies: $1.55\ \mathrm{nA}/\sqrt{f}$. The increase in device area leads to an increase in the 1/f noise current spectral density to: $2.07\ \mathrm{nA}/\sqrt{f}$. It follows that at a frequency of $f = 136$ kHz, the 1/f noise and white noise of the proposed device have equal spectral density.

As a supplement, we further discuss another metric more suitable for comparison, namely wavefront sensitivity, defined as the minimum detectable angle. For an SNR of 35 dB, the detailed derivation is as follows:

Consider three signals Qe_1, Qe_2, Qe_3, when $K_x = K_y = 0$, the means of Qe_1, Qe_2, Qe_3 are all normalized to 1, and the signal-to-noise ratio $SNR = 35dB$, therefore, the noise variance of each signal is:

$$\sigma^2 = 10^{-3.5} \approx 3.162 \times 10^{-4}, \sigma = 0.018.$$

The measurement definitions for the observables M_1 and M_2 are

$$\mathrm{M}_1 = \log_{10} \frac{\mathrm{Qe}_3}{\mathrm{Qe}_1}, \mathrm{M}_2 = \log_{10} \frac{\mathrm{Qe}_2}{\mathrm{Qe}_3}.$$

In the vicinity of $K_x = K_y = 0$, $M_1 = \frac{lnQe_3 - lnQe_1}{ln\,10}, M_2 = \frac{lnQe_2 - lnQe_3}{ln\,10}$, and its covariance matrix is:

$$\Sigma_M = \frac{\sigma^2}{(ln\ 10)^2} \begin{pmatrix} 2 & -1 \\ -1 & 2 \end{pmatrix}$$

Simulation results (Figure 4a, b) show that in the vicinity of $K_x = K_y = 0$, we have:

$$\frac{\partial M_1}{\partial K_x} = 0, \frac{\partial M_1}{\partial K_y} = \frac{36}{7\pi}, \frac{\partial M_2}{\partial K_x} = \frac{\sqrt{3}}{2}\frac{36}{7\pi}, \frac{\partial M_2}{\partial K_y} = -\frac{1}{2}\frac{36}{7\pi}.$$

Then the Jacobian matrix is:

$$\mathbf{J} = \frac{36}{7\pi} \begin{pmatrix} 0 & 1 \\ \frac{\sqrt{3}}{2} & -\frac{1}{2} \end{pmatrix}$$

Thus, the covariance matrix of $\mathbf{K} = [K_x, \mathrm{K_y}]^{\mathrm{T}}$ can be obtained:

$$\Sigma_K = \mathbf{J}^{-1} \Sigma_f (\mathbf{J}^{-1})^{\mathrm{T}}$$

Substituting

$$\mathbf{J}^{-1} = \frac{7\pi}{36}\begin{pmatrix} \frac{1}{\sqrt{3}} & \frac{2}{\sqrt{3}} \\ 1 & 0 \end{pmatrix}$$

into the calculation yields:

$$\Sigma_K = \frac{\sigma^2}{(ln\ 10)^2}\left(\frac{7\pi}{36}\right)^2\begin{pmatrix} 2 & 0 \\ 0 & 2 \end{pmatrix}$$

Therefore, the noise-equivalent error (standard deviation) of K_x, K_y is:

$$\sigma_{K_x} = \sigma_{K_y} = \sqrt{2}\,\frac{\sigma}{ln\,10}\frac{7\pi}{36} = 0.0067$$

Thus, in the vicinity of $K_x = K_y = 0$, the noise-equivalent normalized wave vector component errors in x and y caused by signal noise are approximately 6.67×10^{-3}, and the total noise-equivalent normalized wavefront deviation is $\sqrt{2} \times 6.67 \times 10^{-3} = 9.43 \times 10^{-3}$, corresponding to an incident angle deviation of $9.43 \times 10^{-3}\ rad$. This minimum detectable angle is lower than that of reported device: $9.9 \times 10^{-3}\ rad$ [12] (The understanding of SNR in the references is flawed, specifically, SNR refers to the power ratio of a signal to noise, rather than the absolute value ratio of the signal to noise, resulting in an incorrect decrease of $10^{2.25}$ times in minimum detectable angle).

Note S6. Measurement of the Normalized Wavefront Gradient

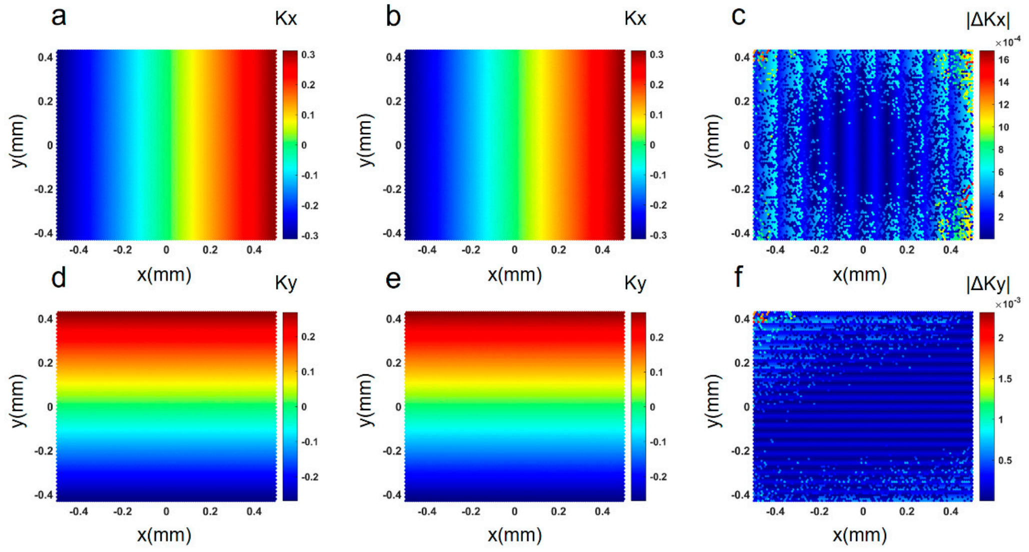

Figure S6. Measurement results of the normalized wavefront gradient for parabolic wavefront. (a) True value of the x-component of the normalized wavefront gradient; (b) Measured value of the x-component of the normalized wavefront gradient; (c) Absolute value of the measurement error for the x-component of the normalized wavefront gradient; (d-f) True value, measured value, and absolute value of the measurement error for the y-component of the normalized wavefront gradient.

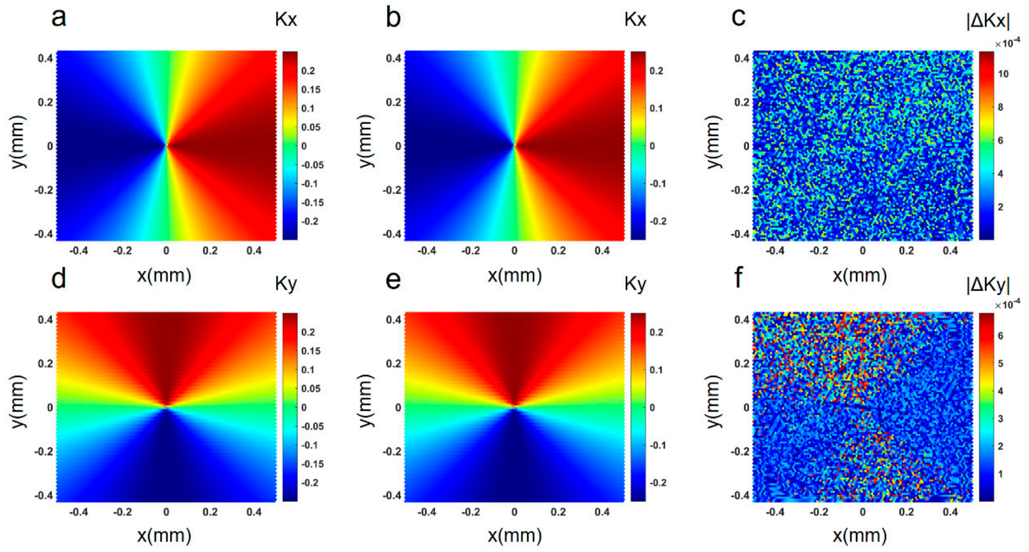

Figure S7. Measurement results of the normalized wavefront gradient for conical wavefront. (a) True value of the x-component of the normalized wavefront gradient; (b) Measured value of the x-component of the normalized wavefront gradient; (c) Absolute value of the measurement error for the x-component of the normalized wavefront gradient; (d-f) True value, measured value, and absolute value of the measurement error for the y-component of the normalized wavefront gradient.

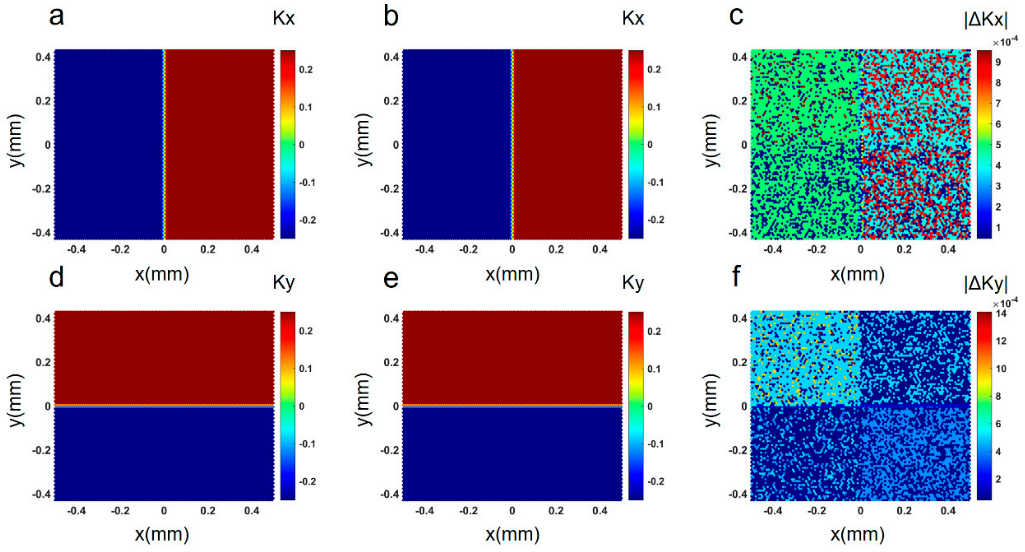

Figure S8. Measurement results of the normalized wavefront gradient for tetrahedral wavefront. (a) True value of the x-component of the normalized wavefront gradient; (b) Measured value of the x-component of the normalized wavefront gradient; (c) Absolute value of the measurement error for the x-component of the normalized wavefront gradient; (d-f) True value, measured value, and absolute value of the measurement error for the y-component of the normalized wavefront gradient.

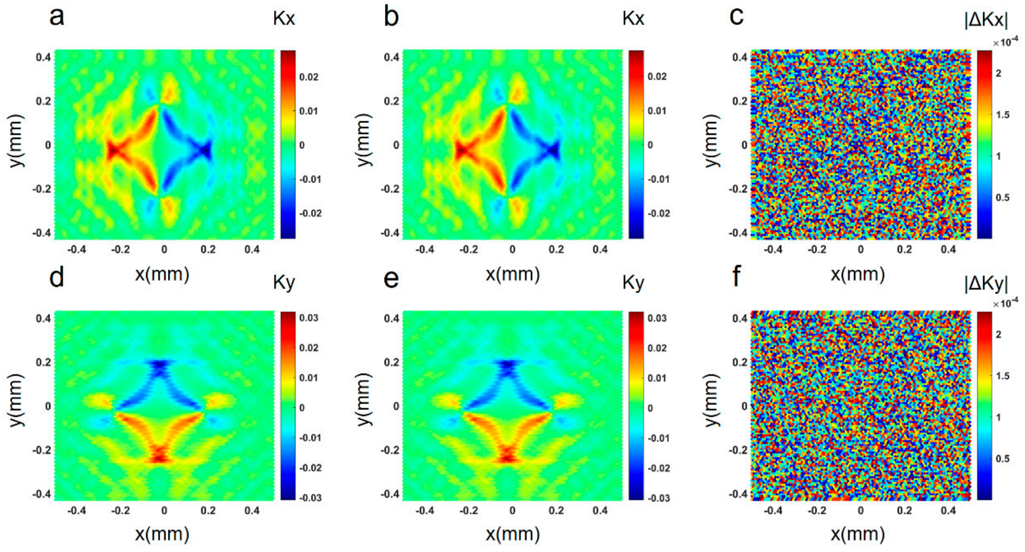

Figure S9. Measurement results of the normalized wavefront gradient for the phase imaging wavefront. (a) True value of the x-component of the normalized wavefront gradient; (b) Measured value of the x-component of the normalized wavefront gradient; (c) Absolute value of the measurement error for the x-component of the normalized wavefront gradient; (d-f) True value, measured value, and absolute value of the measurement error for the y-component of the normalized wavefront gradient.

References for Supplementary Information

Note S7. PC material properties and finite element full wave simulation schematic

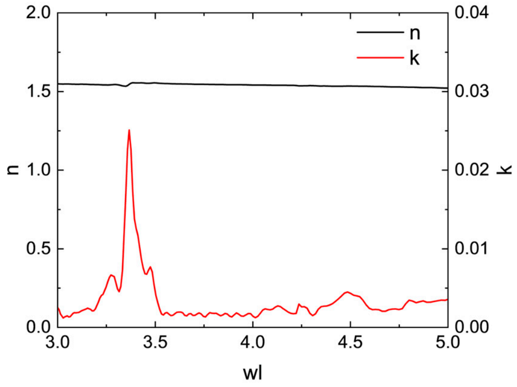

Figure S10. Relationship between the real part n and imaginary part k of the refractive index of PC material and wavelength in the mid-wave infrared band [13].

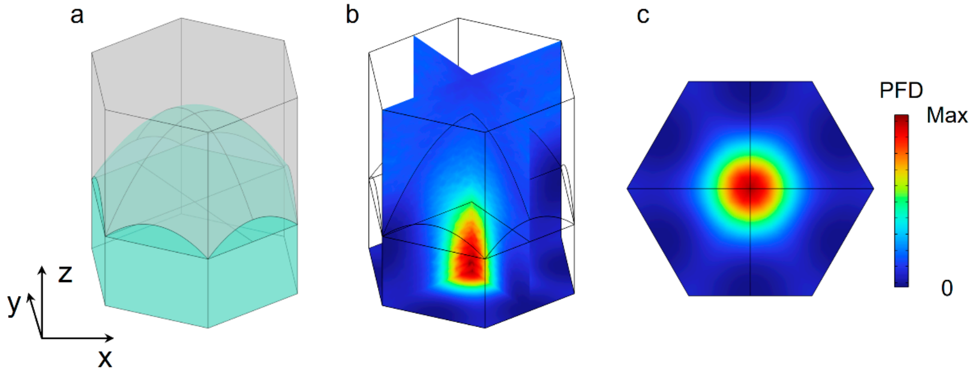

Figure S11. Schematic of full-wave finite-element simulation. (a) Geometric structure diagram of micro lens, the microlenses made of PC material are indicated by the blue part; (b-c) Light focusing capability of the microlens without metal antenna structures, with cross-sectional views showing the time-averaged power flow density distribution along the -z direction.

Note S8. Analysis of the Impact of Fabrication Errors on the Performance of the Proposed Device

The fabrication errors of optoelectronic devices can be broadly categorized into geometric dimension errors and electrical non-uniform contact errors. Among these, the graphene material and metal antenna structures are fabricated using UV lithography and electron-beam lithography, respectively. UV lithography and electron-beam lithography resolutions and overlay accuracies both on the order of tens of nanometers to several nanometers, the errors of this magnitude are negligible in this paper. The most significant geometric fabrication error is expected to arise from the imprint alignment error of the microlenses. The overlay alignment accuracy of current commercial nanoimprint lithography processes is less than 500 nm (NPS300), which will have a certain impact on the device response characteristics. To this end, we simulated the response characteristics of the device under 500 nm alignment offsets in each of two orthogonal directions; detailed results are presented in **Figures S12 and 13**.

Analysis of the results shows that microlens alignment errors at the 500 nm level slightly reduce the angular dynamic range of the proposed device (from 28° to 23°), but do not critically impair the wavefront sensing capability of the proposed device; thus, the error remains within an acceptable range. Electrical non-uniform contact primarily manifests in the contact resistance between graphene and metal antennas. Existing literature reports indicate that the fluctuation range of graphene-metal antenna contact resistance can be as low as approximately ±8% [10]. Owing to the presence of the PC layer, the contact resistance can remain stable over extended periods [14–16]; therefore, contact resistance fluctuations can be corrected through pre-calibration.

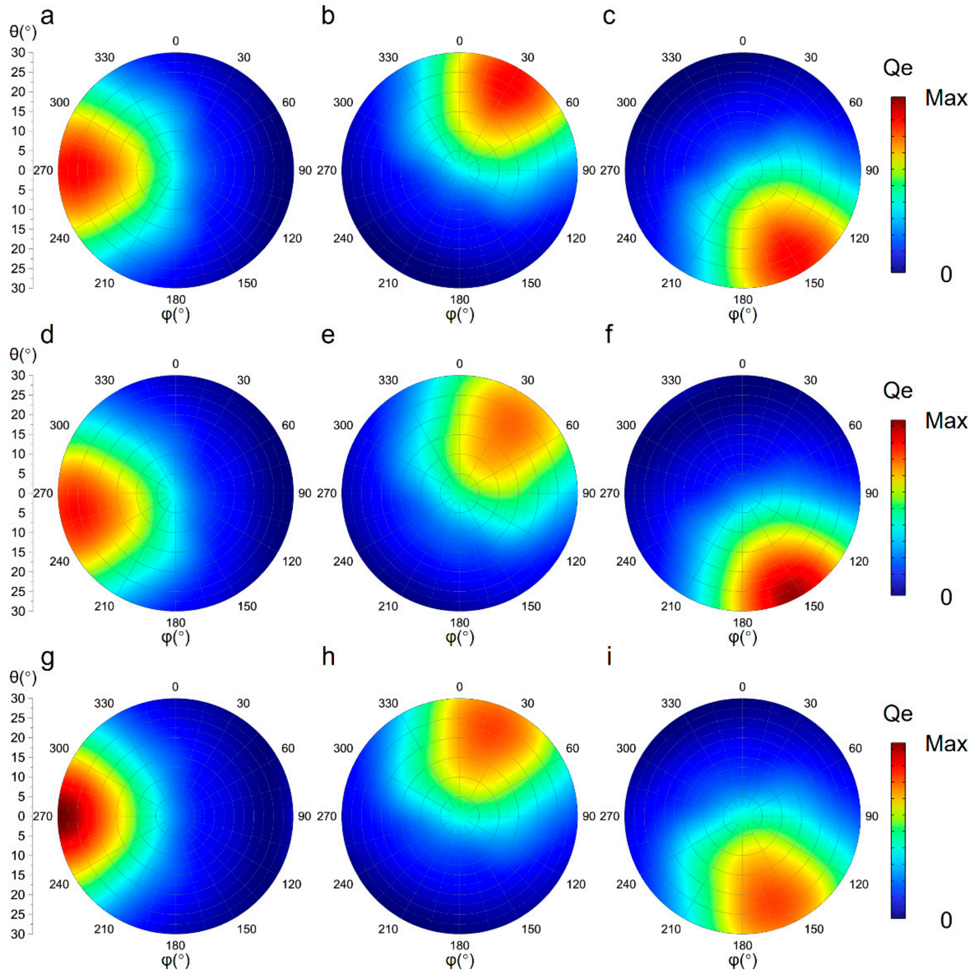

Figure S12. (a-c) Relationship between $Qe_{1\text{-}3}$ and the incident angles θ, φ when the microlens alignment error is 0 nm; (d-f) Relationship between $Qe_{1\text{-}3}$ and the incident angles θ, φ when the microlens alignment error is 500 nm along the φ = 90° direction; (g-i) Relationship between $Qe_{1\text{-}3}$ and the incident angles θ, φ when the microlens alignment error is 500 nm along the φ = 270° direction.

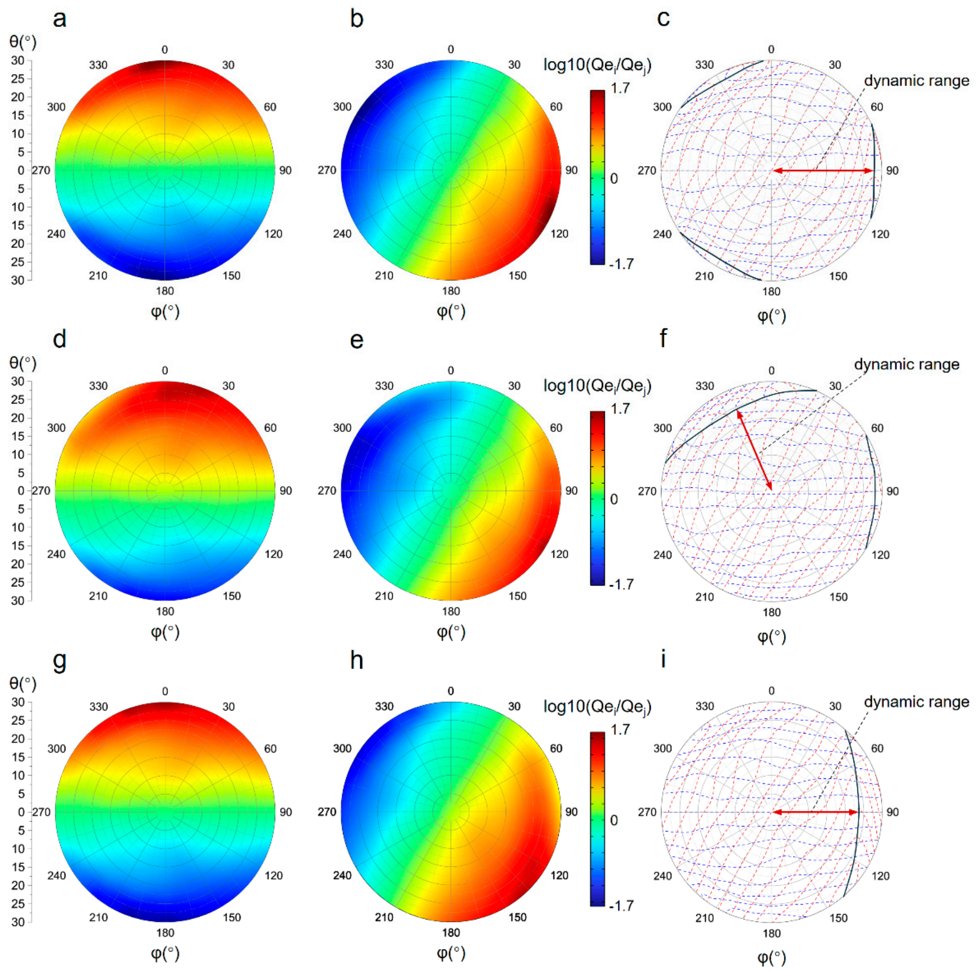

Figure S13. (a-c) Relationship between M_1, M_2 and the incident angles θ, φ when the microlens alignment error is 0 nm, with the dynamic range boundary defined by the black solid line in (c), no less than 28°; (d-f) Relationship between M_1, M_2 and the incident angles θ, φ when the microlens alignment error is 500 nm along the φ = 90° direction, with the dynamic range no less than 24°; (g-i) Relationship between M_1, M_2 and the incident angles θ, φ when the microlens alignment error is 500 nm along the φ = 270° direction, with the dynamic range no less than 23°.

Note S9 Concise Explanation of the Physical Mechanism of the Angular Dynamic Range

Figure S14 illustrates the physical mechanism of the angular dynamic range. (a) shows the time-averaged power flow density distribution on the graphene plane at normal incidence; symmetry protection ensures that the optical absorptions of the three terminals, $Q_{e1\text{-}3}$, remain strictly equal, leading to $M_1 = M_2 = 0$ ($M_1 = log\left(\frac{Q_{e3}}{Q_{e1}}\right), M_2 = log\left(\frac{Q_{e2}}{Q_{e3}}\right)$). However, as the incident angle increases, a scenario such as that described in (c) may occur, where the incident angle θ = 45°, and optical crosstalk from neighboring pixels causes the power flow density distribution within the device to likewise exhibit rotational symmetry, resulting in $Q_{e1\text{-}3}$ being approximately equal, $M_1 \approx M_2 \approx 0$, making it impossible to uniquely determine the incident angle θ from M_1, M_2.

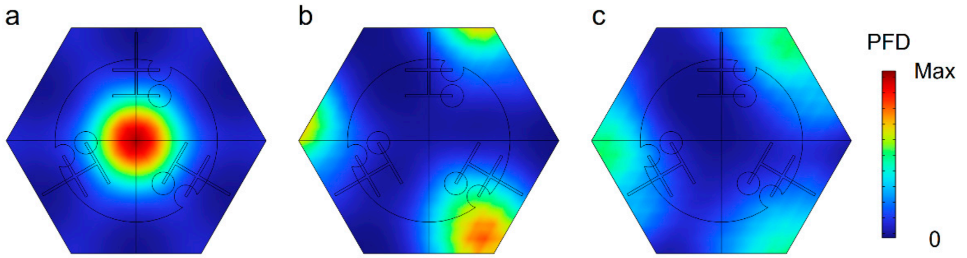

Figure S14. (a) Time-averaged power flow density (PFD) distribution at incident angle θ = 0°; (b) Time-averaged PFD distribution at incident angle θ = 35°; (c) Time-averaged PFD distribution at incident angle θ = 45°.

Note S10 Relationship between Wave Vector Components k_x, k_y, Normalized Wave Vector Components K_x, K_y, and Incident Angles θ, φ

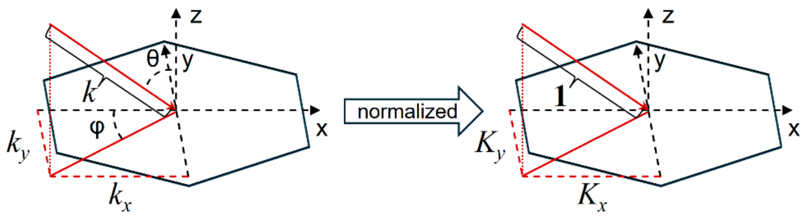

Figure S15. Illustration of the relationship between the wave vector components (k_x, k_y), normalized wave vector components (K_x, K_y), and the incident angles (θ, φ).

1. Dai, X.; Yu, Y.; Ye, T.; Deng, J.; Bu, Y.; Shi, M.; Wang, R.; Zhou, J.; Sun, L.; Chen, X.; et al. Dynamically Reconfigurable On-Chip Polarimeters Based on Nanoantenna Enabled Polarization Dependent Optoelectronic Computing. *Nano Lett.* **2024**, *24*, 983–992, doi:10.1021/acs.nanolett.3c04454.
2. Liu, X.; Chen, Y.; Kong, X.; Liu, W.; Ni, Z.; Lu, J.; Wang, Q.J.; Lee, C.; Wei, J.; Qiu, C.-W. Metaphotonic Photodetectors for Direct Stokes Quantification. *Nat Electron* **2025**, *8*, 1099–1107, doi:10.1038/s41928-025-01481-4.
3. Wei, J.; Xu, C.; Dong, B.; Qiu, C.-W.; Lee, C. Mid-Infrared Semimetal Polarization Detectors with Configurable Polarity Transition. *Nat. Photon.* **2021**, *15*, 614–621, doi:10.1038/s41566-021-00819-6.
4. Haynes, W.M. *CRC Handbook of Chemistry and Physics*; CRC press, 2016;
5. Rut'kov, E.V.; Afanas'eva, E.Y.; Gall, N.R. Graphene and Graphite Work Function Depending on Layer Number on Re. *Diamond and Related Materials* **2020**, *101*, 107576, doi:10.1016/j.diamond.2019.107576.
6. Das Sarma, S.; Adam, S.; Hwang, E.H.; Rossi, E. Electronic Transport in Two-Dimensional Graphene. *Rev. Mod. Phys.* **2011**, *83*, 407–470, doi:10.1103/RevModPhys.83.407.
7. Southwell, W.H. Wave-Front Estimation from Wave-Front Slope Measurements. *J. Opt. Soc. Am.* **1980**, *70*, 998, doi:10.1364/JOSA.70.000998.
8. Piotrowski, J.; Rogalski, A. *High-Operating-Temperature Infrared Photodetectors*; SPIE press monograph; SPIE press: Bellingham, Wa, 2007; ISBN 978-0-8194-6535-1.
9. Blecha, T.; Vlčková Živcová, Z.; Sonia, F.J.; Mergl, M.; Volochanskyi, O.; Bodnár, M.; Rous, P.; Mizohata, K.; Kalbáč, M.; Frank, O. Electrical Contact Resistance of Large-Area Graphene on Pre-Patterned Cu and Au Electrodes. *Nanomaterials* **2022**, *12*, 4444, doi:10.3390/nano12244444.
10. Popescu, S.M.; Barlow, A.J.; Ramadan, S.; Ganti, S.; Ghosh, B.; Hedley, J. Electroless Nickel Deposition: An Alternative for Graphene Contacting. *ACS Appl. Mater. Interfaces* **2016**, *8*, 31359–31367, doi:10.1021/acsami.6b08290.
11. Paladino, E.; Galperin, Y.M.; Falci, G.; Altshuler, B.L. 1/f Noise: Implications for Solid-State Quantum Information. *Rev. Mod. Phys.* **2014**, *86*, 361–418, doi:10.1103/RevModPhys.86.361.
12. Yi, S.; Xiang, J.; Zhou, M.; Wu, Z.; Yang, L.; Yu, Z. Angle-Based Wavefront Sensing Enabled by the near Fields of Flat Optics. *Nat Commun* **2021**, *12*, 6002, doi:10.1038/s41467-021-26169-z.
13. Zhang, X.; Qiu, J.; Li, X.; Zhao, J.; Liu, L. Complex Refractive Indices Measurements of Polymers in Visible and Near-Infrared Bands. *Appl. Opt.* **2020**, *59*, 2337, doi:10.1364/AO.383831.
14. Zhou, G.; Li, Z.; Ge, Y.; Zhang, H.; Sun, Z. A Self-Encapsulated Broadband Phototransistor Based on a Hybrid of Graphene and Black Phosphorus Nanosheets. *Nanoscale Adv.* **2020**, *2*, 1059–1065, doi:10.1039/C9NA00528E.
15. Bennison, M.J.; Collins, A.R.; Zhang, B.; Evans, R.C. Organic Polymer Hosts for Triplet–Triplet Annihilation Upconversion Systems. *Macromolecules* **2021**, *54*, 5287–5303, doi:10.1021/acs.macromol.1c00133.

16. Sønstevold, L.; Czerkies, M.; Escobedo-Cousin, E.; Blonski, S.; Vereshchagina, E. Application of Polymethylpentene, an Oxygen Permeable Thermoplastic, for Long-Term on-a-Chip Cell Culture and Organ-on-a-Chip Devices. *Micromachines* **2023**, *14*, 532, doi:10.3390/mi14030532.
